# Evidence of validity and reliability of a scale to evaluate gratitude toward the organization in the Peruvian context

**DOI:** 10.3389/fpsyg.2025.1421726

**Published:** 2025-09-10

**Authors:** Edgardo Muguerza-Florián, Elizabeth Emperatriz García-Salirrosas, Miluska Villar-Guevara, Israel Fernández-Mallma

**Affiliations:** ^1^UPG de Ciencias Empresariales, Escuela de Posgrado, Universidad Peruana Unión, Lima, Peru; ^2^Faculty of Management Science, Universidad Autónoma del Perú, Lima, Peru; ^3^EP de Administración, Facultad de Ciencias Empresariales, Universidad Peruana Unión, Juliaca, Peru; ^4^EP de Educación, Facultad de Ciencias Humanas y Educación, Universidad Peruana Unión, Juliaca, Peru

**Keywords:** gratitude toward the organization, dimensionality, validation, psychometric properties, Peru

## Abstract

**Introduction:**

Despite gratitude's potential to promote wellbeing and performance in organizational settings, its role in the workplace is understudied. Although the literature indicates few attempts to develop scales for evaluating this construct in North America, no Spanish version exists to assess the validity and reliability of these scales in other contexts. This study aimed to translate into Spanish, adapt, and analyze the validity and reliability of a scale to evaluate gratitude toward the organization in Peruvian teachers.

**Methods:**

An instrumental study was carried out through convenience sampling. A sample of 448 Regular Basic Education teachers from a private educational network that has an important presence in the three regions of Peru was used. The analyses were performed using the Covariance Structural Equation Model (CB-SEM) with the AMOS 24 statistical software.

**Results:**

Confirmatory Factor Analysis provided model fit indices (2 factors) between excellent and acceptable (CMIN/DF = 2.872; CFI = 0.983; SRMR = 0.028; RMSEA = 0.064; Pclose = 0.031). It also demonstrated good internal consistency (α = 0.890 and 0.953; CR = 0.895 and 0.954; AVE = 0.682 and 0.721).

**Discussion:**

The results of this study support this measurement scale as a two-dimensional tool that can be used in business practice to measure gratitude in organizations and study the effectiveness of interventions related to wellbeing and gratitude in the workplace.

## 1 Introduction

Gratitude is considered a predominant social phenomenon analyzed from an emotional and interpersonal perspective (Wnuk, 2020). From a general approach, gratitude is considered an emotion that drives people to value a job or a good action that another has done or is willing to do and pay in some way (Deluca et al., 2023; de Medeiros et al., 2023; Garg, 2023b). Other studies define it as knowing how to be kind to another who demonstrated the same attitude (Adair et al., 2020; Wnuk, 2020). According to this definition, gratitude is a feeling that arises from the desire to return the favor received, which must be reciprocated in some way, and it is an emotion that arises from the recognition of the kindness received (Cain et al., 2019; Cortini et al., 2019). In the field of psychology, gratitude is treated as an emotion (Maslow, 1968), a moral attitude, a strength of character (Park et al., 2004), a personality trait, or a stress coping mechanism (McCullough et al., 2002). Positive psychology views gratitude as a character strength (Park et al., 2004) and that, along with hope, creates enthusiasm, appreciation for beauty, humor, spirituality, and forgiveness. Furthermore, it projects the sense of transcendence (Scorsolini-Comin et al., 2013).

Gratitude as a moral emotion plays the role of a moral barometer because it “measures” the level of effort and value of the grantor's favor. It serves as a moral motivator because it encourages the recipient to reciprocate the good received. Moreover, it serves as a moral reward because it increases the probability of helping the recipient in the future and positively influences strengthening that behavior in the grantor (McCullough et al., 2001). Some have also seen gratitude as a moral norm (Buksik, 2002) because it has become a specific form of generalized social obligation, formed based on situations and experiences that individuals go through during their lives, which have led them to believe that they need to be grateful in certain situations. Gratitude is seen as a moral emotion (Maslow, 1991). It provides another, much more profound and transcendental component, that of the awareness of existence as a gift. Regardless of the source from which that consciousness comes, which could be religious or mystical, the fact of knowing oneself is special, perceiving life as a gift, experiencing admiration, respect, adoration toward the other person, the past, the world, nature, divinity or that which contributed to their current state, triggers a desire to reciprocate the gift received (Wnuk, 2020). Because in many religious systems gratitude is considered a desired virtue and of a very high level, studies on public and private manifestations of religiosity have been positively related to the disposition to be grateful and with the feeling of gratitude (Emmons and Crumpler, 2000).

In the organizational field, gratitude is expressed by employees toward their organization (Wnuk, 2020). It encompasses job rewards, professional growth, fair treatment, and the feeling that one's employer provides opportunities for growth and promotion. Research has examined how gratitude can modify employees' attitudes by providing social support or awakening altruism in the workplace, and its association with efficiency, productivity, and work performance (Grant and Gino, 2010). Additionally, it has explored how gratitude affects organizational citizenship behaviors. Gratitude is also an important indicator of an organization's health and wellbeing (Di Fabio et al., 2017) and can be measured individually and collectively (Fehr et al., 2017).

Gratitude has been conceptualized in the organizational field at three levels: event level, which corresponds to episodic gratitude, individual level to persistent gratitude, and organizational level to collective gratitude (Fehr et al., 2017; Souza et al., 2020). Studies have also been conducted on the association between gratitude, anxiety, and error management in organizations (Wang et al., 2020), as well as their impact on wellbeing, performance, and organizational citizenship behavior (Sekaja et al., 2022). Studies have shown that gratitude can contribute to prosocial motivation in volunteers, stimulates positive emotions, and in the organizational context promotes the wellbeing and retention of volunteers, as well as the development of personal resources (Yang et al., 2023). Systematic reviews have attempted to integrate the various approaches to gratitude research, revealing that the field of study still requires further exploration (Locklear et al., 2023).

Various scales of gratitude oriented toward religion have been constructed (Garg et al., 2021; Garg, 2023a,b), gratitude linked to resentment and appreciation (Garg and Mehak, 2023), gratitude scale in Brazilian adults (Deluca et al., 2023), gratitude seen from positive psychology associated with subjective well-being (Vazquez et al., 2020), another gratitude questionnaire has been validated that seeks to understand the predictors and consequences of gratitude (de Medeiros et al., 2023). Validations and adaptations of the Gratitude Questionnaire−20 Items (G20) to English have also been carried out (Bernabe-Valero et al., 2020). In this sense, gratitude in the organizational sphere has been identified as a key element for fostering wellbeing, commitment, and promoting healthy relationships at work. For this purpose, a few psychometric instruments have been created to measure this construct at both the organizational and personal levels. One of the most common instruments is the Gratitude Questionnaire-6 (GQ-6), a metric that has demonstrated good internal consistency and has been validated in diverse cultural realities. However, the GQ-6 approaches gratitude from a general perspective, without considering the specific organizational environment or daily work interactions, which restricts its usefulness in measuring gratitude toward the organization as a contextualized phenomenon (McCullough et al., 2002). Another frequently used metric is the Gratitude at Work Scale (GAWS), which examines two important aspects: gratitude toward a supportive work environment and gratitude for performing meaningful work. This scale has demonstrated good internal consistency (Cain et al., 2019). Furthermore, the most recent is the Work Gratitude Scale (WGS), a multidimensional tool that encompasses grateful appreciation, gratitude toward others, and a proactive attitude. This metric has been tested and shows strong indicators of reliability (Youssef-Morgan et al., 2022).

While studies on gratitude have been conducted in countries such as the United States, Italy, Chile, France, Hong Kong, Japan, Poland, Singapore, South Africa, and South Korea, the instruments used in these studies have primarily been in non-Latin American contexts and sectors unrelated to education, highlighting the need for culturally relevant tools validated in contexts with specific characteristics. This is particularly critical in the Peruvian educational setting, where gratitude toward the organization can present itself with specific characteristics of teachers' work environments. Therefore, translating into Spanish, adapting, and analyzing the validity and reliability of an organizational gratitude scale for the Peruvian context will be a significant contribution, as currently there is no instrument with adequate psychometric properties for this population and field of study. In this sense, this could represent a valuable resource for evaluating this construct and, in the future, designing studies that allow for suggesting improvement policies in Peruvian entities.

## 2 Literature review

### 2.1. Gratitude toward the organization

In terms of positive psychology, experts in the area have defined gratitude as a dispositional tendency, known as a human condition (Palazzeschi et al., 2022). Some define gratitude as part of a broader life orientation aimed at understanding and appreciating the good in the world (McCullough et al., 2001; Cain et al., 2019). Some studies have shown that greater dispositional gratitude increases the tendency to engage in positive meditation, which in turn affects the interpretation of life events (Cortini et al., 2019). In short, gratitude is described with affective and cognitive elements (Chen et al., 2009), and the interest in translating it as an organizational value capable of positively influencing individual and collective dimensions is relatively recent (Komase et al., 2020). Some relevant scientific contributions have found that active gratitude is not associated with a contractual relationship or a specific job, but with a personality trait (Sudiro et al., 2023).

In the organizational context, gratitude can be approached from three levels: the individual, event, and organizational levels (Wang et al., 2020). A recent contribution by Di Fabio et al. (Di Fabio et al., 2017) related to this construct represents its position in the context of positive psychology and the psychology of sustainability of harmony, collecting and analyzing various contributions from the literature to explain the importance of gratitude in various aspects of organizational life. Furthermore, some studies reveal its strong association with psychological welbeing, confidence, and commitment (Chou and Chen, 2018), stress and burnout (Maghsoudi, 2020), and hope and optimism (Adair et al., 2020). Furthermore, it has been shown how much gratitude impacts the mediating effects of psychological exhaustion in its link with work stress (Hwang and Lee, 2021).

In this sense, there are many socially acceptable ways to express gratitude at work (Williams, 2023). In light of this, team leaders need to let their members know that they trust them and that they are proud of their work, avoiding micromanagement and looking for more opportunities to praise them (Emmons and Crumpler, 2000; Zheng et al., 2024). Sincere feelings of gratitude, along with the willingness to help and the courage to act accordingly, show how much your work impacts the institutional mission and how your efforts serve the community (Bernabe-Valero et al., 2020; Locklear et al., 2023; Yang et al., 2023).

On the other hand, people who can express gratitude in their own lives do not necessarily do so in work environments (Cain et al., 2019). It is also said that the results of some studies related to this topic, such as job performance, are positively associated with gratitude toward the organization (Cain et al., 2019; Komase et al., 2020; Wang et al., 2020). In an organizational context, gratitude can be seen as an expression of appreciation for the benefits associated with work and how these benefits contribute to people's lives (Cain et al., 2019; Unanue et al., 2021), that is, a person's ability to experience positive emotions at work. Finally, having demonstrated the benefits of gratitude, organizations can use it as a strategy to improve employee well-being (Di Fabio et al., 2017; Fehr et al., 2017; Youssef-Morgan et al., 2022; Zheng et al., 2024).

### 2.2. Analysis of the scales that evaluate gratitude toward the organization

The literature offers many scales to measure gratitude, most of them multidimensional. However, these do not focus on an evaluation of gratitude in organizational contexts (Watkins et al., 2003; Adler and Fagley, 2005; Morgan et al., 2017). Some studies on gratitude toward the organization have been developed under a qualitative approach (Sekaja et al., 2022; Williams, 2023), while others have observed this construct from the quantitative approach (Di Fabio et al., 2017; Cortini et al., 2019; Wang et al., 2020; Unanue et al., 2021; Palazzeschi et al., 2022). In this sense, it is worth highlighting that a recent study applied to a total of 1,575 American health workers (clinical and non-clinical), examined the effectiveness of a gratitude letter intervention as an effective tool to improve emotional exhaustion, wellbeing, and the balance between work and personal life of health professionals (Adair et al., 2020). Awakening the interest of academia in evaluating the behavior of this construct in business environments.

In this sense, it is important to have valid and reliable tools that can be used in the different realities of business life. Given that gratitude toward the organization is a clear indicator of a positive work environment, an instrument to assess this construct is necessary for several reasons. When employees feel grateful for the opportunities, support, and work environment the company provides them, they feel more motivated, committed, and satisfied with their work. By measuring gratitude toward the organization, one can identify the aspects of organizational culture and company policies that contribute to this sense of appreciation and connection, and how this affects employee performance, enabling companies to adjust and improve their strategies. Additionally, it could help identify opportunities to foster this employee cooperation and cohesion further. Next, an analysis of the latest measurement scales published in high-impact journals will be presented:

The Gratitude Questionnaire−6 (GQ-6) was developed in the USA by McCullough et al. (2002). This unidimensional scale was applied to 238 participants. This is a self-report instrument that is made up of 6 items with a seven-point Likert scale rating (where 1 is “strongly disagree” and 7 is “strongly agree”). Their study suggests that gratitude as an emotional state is related to, but distinct from, other specific emotional states (hope, depression, anxiety, jealousy, happiness, and energy) and is a specific example of positive emotions such as happiness, joy, and satisfaction. For internal consistency estimates, Cronbach's Alpha (α = 0.82) was used. The results demonstrated a scale with good psychometric properties. Later studies used the GQ-6 as evidence of its validity (Wang et al., 2020; Unanue et al., 2021; Zheng et al., 2024).

The Gratitude at Work Scale (GAWS) was developed and validated in the USA by Cain et al. (2019). This two-dimensional scale (Gratitude for Supportive Work Environment and Gratitude for Meaningful Work) was applied to 207 full-time employees belonging to various human services and mental health agencies. This is a self-report instrument that is made up of ten items with a five-point Likert scale rating (where 1 is “never” and 5 is “almost always”). For internal consistency estimates, Cronbach's Alpha was used (α = 0.83 and 0.83). The results demonstrated a scale with good psychometric properties. Subsequently, Komase et al. (2020) evaluated the reliability and validity of the Japanese version, reporting good internal consistency (α =0.81 and 0.91). In this sense, the GAWS has psychometric support and can be useful to investigate functioning and wellbeing in the workplace.

The Work Gratitude Scale (WGS) was developed in the USA by Youssef-Morgan et al. (2022). This unidimensional scale was applied to 625 employees in various positions. This self-report instrument comprises 10 items across 3 dimensions: grateful appraisals, gratitude toward others, and an intentional attitude of gratitude. The rating was a seven-point Likert-type response scale (where 1 is “strongly disagree” and 7 is “strongly agree”). To determine the level of internal consistency, three indicators were calculated: point estimate composite reliability (ρ = 0.82), McDonald's Omega (ω = 0.82), and Cronbach's Alpha (α = 0.82). The findings of this study support the WGS as a multidimensional measurement tool that can be employed in various business settings to assess overall gratitude and track the effectiveness of gratitude-related activities in the workplace.

## 3 Materials and methods

### 3.1 Design

Research with an instrumental design in order to review the psychometric properties of the instrument of gratitude toward the organization to demonstrate its usefulness (Ato et al., 2013).

### 3.2 Sample

The population consisted of Regular Basic Education teachers (preschool, primary, and secondary). This study considered the participation of various educational institutions belonging to a network of private schools in Peru. This educational system holds significant importance in the region, and its educational model and philosophy apply to various similar institutions in South America. Academics are increasingly interested in its management model and in addressing high-impact topics, especially applicable to educational institutions.

Prior to the application, the research proposal was approved by the board of the Ethics Committee of the Graduate School of a private University in Peru (2023-CE-EPG-00034). Among the inclusion criteria, it was considered that the participating teachers had to be working for an educational institution in the network at the time of the questionnaire application. Informed consent and assent were obtained from the educational network through its representative leaders. The questionnaire was administered during the first half of 2023. Using non-probabilistic convenience sampling, the survey was administered through a virtual link, and the questionnaire was hosted on Google Forms. This questionnaire was self-administered and distributed by email to a total of twenty eight educational institutions in the network. A total of four hundred and forty eight valid surveys were taken into account, in which the participating teachers offered their answers voluntarily (Table 1).

**Table 1:** Table 1 Sociodemographic characteristics of the participants (*n* = 448).

**Age range**	**Sex**				**Total**	
	**Female**		**Male**			
	***n***	**%**	***n***	**%**	***n***	**%**
Under 29	69	15.4	36	8.0	105	23.4
30–39	100	22.3	fifty	11.2	150	33.5
40–49	76	17.0	37	8.3	113	25.2
50 or more	42	9.4	38	8.5	80	17.9
Total	287	64.1%	161	35.9%	448	100.0%
**Geographic location**			**Teaching level**			
	***n***	**%**			***n***	**%**
Coast	262	58.5	Initial		54	12.1
Jungle	120	26.8	Primary		196	43.8
Mountain range	66	14.7	Secondary		198	44.2
Total	448	100.0	Total		448	100.0

### 3.3 Instrument

The Gratitude Toward the Organization scale was designed in Poland by Wnuk (2020) and considered a short scale of only 12 items. During the construction of this scale, the most important characteristics of the study variables were considered. Psychometric properties supported a two-factor scale: (1) gratitude as obligation to reciprocity (COM) and (2) gratitude as morality (MOR). The method used to develop the instrument is a Likert-type response format with five response options: “I totally disagree”, “I do not agree”, “It is difficult to say”, “Yes”, “I agree”, and “I totally agree” on a scale from 1 to 5. The reported values of Cronbach's Alpha for the internal reliability of the instrument, in the original study sample, ranged between 0.890 and 0.953.

### 3.4 Translation process

The original English version of the Gratitude Toward the Organization Scale was translated into Spanish using a bilingual back-translation method. Two bilingual native speakers of Spanish (Spanish-English) independently completed the translation of the scale. A focus group of six faculty members (according to the study's inclusion criteria) compared, discussed, and revised the translations, resulting in the first draft of the Spanish questionnaire. Using the same method, two bilingual English speakers translated the questionnaire from Spanish to English. The English and Spanish versions of the Organizational Gratitude Scale were tested bilingually on the target population and distributed to the study sample with some final revisions.

### 3.5 Data analysis procedure

Two statistical programs were used to analyze the data: The first was the SPSS software version 25, for the descriptive analysis that would allow the sociodemographic characteristics of the teachers to be evaluated, and that would also serve for the Exploratory Factor Analysis (EFA). The second statistical program was used for covariance structural equation modeling (CB-SEM), requiring the use of AMOS version 24 software, in order to carry out Confirmatory Factor Analysis, evaluate convergent and discriminant reliability, and adjust the measurement model. This is a recommended method to estimate and analyze the validity and reliability of measurement models (Fornell and Larcker, 1981).

## 4 Result

To identify the factorial condition of the scale, an Exploratory Factor Analysis (EFA) was carried out on each element, observing that the items are assigned into two factors based on the construct examined (Table 2). The difference is quite clear between the two factors. The KMO and Bartlett test (Kaiser-Meyer-Olkin correlation coefficient = 0.944) has a value greater than 0.7, and the Bartlett test (Sig = 0.000) is very significant for carrying out factor analysis. The total variance specified in the model is 70.758%, exceeding 50%. The appreciation is contributed by compensation (COM) at 63.240% and by moral values (MOR) at 7.518%. Each item of the original scale was grouped according to its dimensions. Subsequently, Confirmatory Factor Analysis (CFA) was carried out.

**Table 2:** Exploratory Factor Analysis (EFA).

**Pattern matrix**		
**Code**	**Factor**	
	**1**	**2**
COM3	0.964	
COM2	0.898	
COM4	0.891	
COM6	0.847	
COM1	0.788	
COM8	0.771	
COM7	0.731	
COM5	0.661	
MOR2		0.963
MOR1		0.771
MOR4		0.705
MOR3		0.676

Extraction method: maximum likelihood. Rotation method: Promax with Kaiser normalization.

To. The rotation converged in 3 iterations.

The validation of the final measurement model with convergent reliability and validity is evident in Table 3. The Cronbach's Alpha (α) values were 0.953 and 0.890, considered satisfactory values since all levels of this coefficient must be above 0.70. for the model to be valid (Agbo, 2010). Furthermore, the reliability values (CR) were 0.954 and 0.895, which is favorable because this value must be greater than 0.70 to be considered a perfect model (Bagozzi and Yi, 1988). Likewise, the AVE values showed 0.721 and 0.682, which are considered acceptable since this index must be equal to or greater than 0.50 (Hair et al., 2014). In that sense, these values translate as an acceptable measurement model that meets favorable levels of reliability and convergent validity.

**Table 3:** Validation of the measurement model and convergent validity.

**Predictor**	**Code**	**Std beta**	**Cronbach's alpha**	**CR**	**AVE**
Gratitude as an obligation to reciprocity (COM)	COM8	0.864^***^	0.953	0.954	0.721
	COM7	0.808^***^			
	COM6	0.864^***^			
	COM5	0.720^***^			
	COM4	0.922^***^			
	COM3	0.909^***^			
	COM2	0.841^***^			
	COM1	0.850^***^			
Gratitude as morality (MOR)	MOR4	0.725^***^	0.890	0.895	0.682
	MOR3	0.849^***^			
	MOR2	0.879^***^			
	MOR1	0.842^***^			

Cronbach's alpha (α) for all variables is > 0.80, the composite reliability (CR) > 0.80, and the Mean-Variance Extracted (AVE) > 0.60;

*** *p* < 0.001 (significance level), indicating a significant validity of the model.

**Figure 1:**
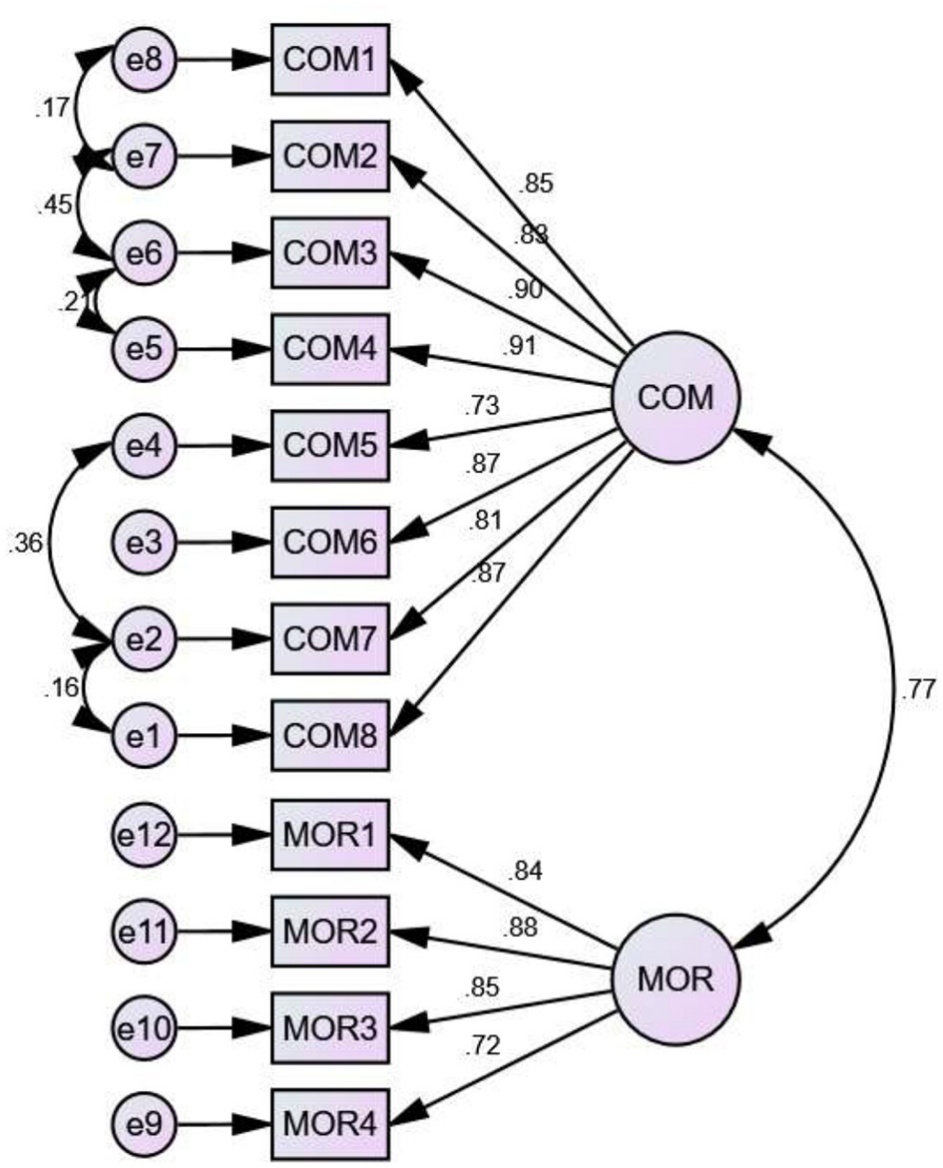
Measurement model of the gratitude towards the organization scale.

What is shown in Table 4 are the goodness-of-fit indicators of the measurement model of the Gratitude Toward the Organization scale. The reported findings from the CFA of a two-dimensional model showed that the 12 items of the scale explained these two factors (Model 1). However, not all goodness-of-fit indicators were excellent; For this reason, the model was re specified based on the Modification Index (MI) (Brown, 2003). In that sense, due to similar wording of the items, there were correlations between the errors of some of them (Figure 1). In this way, the measurement model was analyzed by correlating the errors in the following way: e1 with e2, e2 with e4, e5 with e6, e6 with e7, and e7 with e8 (Model 2), obtaining fit indices between excellent and acceptable.

**Table 4:** Statistical goodness-of-fit indices of the gratitude scale toward the organization in the Peruvian context (Own elaboration).

**Measure**	**Threshold**	**Model 1**		**Model 2**	
		**Estimate**	**Interpretation**	**Estimate**	**Interpretation**
CMIN	–	318.552	–	137.861	–
DF	–	53.000	–	48.000	–
CMIN/DF	Between 1 and 3	6.010	Terrible	2.872	Excellent
CFI	>0.95	0.948	Acceptable	0.983	Excellent
SRMR	< 0.08	0.037	Excellent	0.028	Excellent
RMSEA	< 0.06	0.105	Terrible	0.064	Acceptable
PClose	>0.05	0.000	Not estimated	0.031	Acceptable

According to Fornell and Larcker (1981), the discriminant validity of the measurement model is validated whenever the square root of the AVE of a construct is greater than the values of its correlations with another construct. Table 5 shows that the square root values of both dimensions are greater than their correlation; therefore, the discriminant validity of the measurement model is evident.

**Table 5:** Validation of the discriminant validity of the measurement model.

**Predictor**	**CR**	**AVE**	**COM**	**MOR**
**COM**	0.953	0.719	**0.848**	
**MOR**	0.895	0.682	0.771^***^	**0.826**

*** *p* < 0.001 (significance level). The diagonal values in bold represent the square of the Average Variance Extracted (AVE).

Finally, the final version of the instrument, which underwent rigorous content validity, EFA, and CFA processes to ensure reliable psychometric properties for use, is described (Table 6). It is made up of two factors: 08 items for (1) gratitude as obligation to reciprocity (COM) and 04 items for (2) gratitude as morality (MOR).

**Table 6:** Final instrument of 12 items in Spanish with an English translation.

**Predictor**	**Measurement ítems**	**Affirmations**
Gratitude as an obligation to reciprocity (COM)	COM1	Me siento agradecido con mi organización/empresa. *(I feel grateful to my organization/company)*.
	COM2	He aprendido mucho trabajando en esta organización/empresa. *(I have learned a lot working in this organization/company)*.
	COM3	He adquirido muchas experiencias valiosas mientras trabajaba aquí. *(I have gained many valuable experiences while working here)*.
	COM4	Me alegro de haber llegado a una organización/empresa tan agradable. *(I am glad to have come to such a nice organization/company)*.
	COM5	Me siento en deuda con la organización por todo lo que he recibido de ella. *(I feel indebted to the organization for everything I have received from it)*.
	COM6	Agradezco a la organización/empresa la posibilidad de conocer muchas personas valiosas. *(I thank the organization/company for the possibility to meet many valuable people)*.
	COM7	He recibido mucho de mi trabajo en esta organización/empresa. *(I have received a lot from my work in this organization/company)*.
	COM8	Me siento orgulloso de trabajar en esta organización/empresa. *(I am proud to work in this organization/company)*.
Gratitude as morality (MOR)	MOR1	Considero que todos deberían estar agradecidos por lo que reciben de la organización/empresa, aunque no siempre sea lo que se espera. *(I believe that everyone should be grateful for what they receive from the organization/company, even if it is not always what is expected)*.
	MOR2	La gratitud hacia la organización/empresa es una reacción natural y normal, independientemente de las circunstancias. *(Gratitude towards the organization/company is a natural and normal reaction, regardless of the circumstances)*.
	MOR3	Uno debe estar agradecido por todo en la vida. *(One should be grateful for everything in life)*.
	MOR4	No entiendo a las personas que son desagradecidas con sus empleadores, porque creo que todos merecen experimentar la gratitud. *(I do not understand people who are ungrateful to their employers, because I believe that everyone deserves to experience gratitude)*.

## 5 Discussion

The present study aimed to adapt and analyze the validity and reliability of the Gratitude Toward the Organization Scale originally constructed by Wnuk (2020). As the first study adapted to the Peruvian context and the Spanish language, it represents a crucial first step in awakening interest in this field of study in this region of the world. The results of the present study support the validity and reliability of a bifactorial structure that addresses gratitude toward the organization in two theoretical dimensions, gratitude as obligation to reciprocity (COM) and gratitude as morality (MOR); both have solid psychometric indicators (α > 0.89; CR > 0.89; AVE > 0.68). These findings are consistent with the multidimensional approach presented by Youssef-Morgan et al. (2022), who conceptualized gratitude in the workplace as a second-order construct, composed of three dimensions (grateful appraisals, gratitude toward others, and intentional attitude of gratitude). Both studies agree in characterizing gratitude not as a fixed trait, but as an adaptable psychological resource, found within particular contexts, and that can be deliberately fostered. However, while the model proposed by Youssef-Morgan et al. (2022) emphasizes affective, cognitive, social, and intentional aspects, current research highlights normative and relational factors that are representative of a collectivist work culture. In this environment, the principles of reciprocity, and interpersonal ethics play a fundamental role in the experience of gratitude in work environments. This difference could indicate cultural variations in how gratitude is understood in organizations, highlighting the importance of adapting and validating gratitude scales according to the specific sociocultural context where they are implemented.

Furthermore, the study's findings partially align with those of Cain et al. (2019), who developed the Gratitude at Work Scale (GAWS) as a two-factor tool measuring Gratitude for a Supportive Work Environment (GAWS-SWE) and Gratitude for Meaningful Work (GAWS-MW), also achieving good internal consistency and model fit indices (α > 0.83; CFI > 0.95; RMSEA > 0.08; SRMR > 0.07). They designed a metric focused on dimensions more related to experiences and situations (context and the meaning of work). Thus, although both scales highlight the importance of contextualizing gratitude in the workplace, the adaptation made here suggests that gratitude towards the organization could have different meanings depending on the sociocultural context, which underscores the need to create tools that are culturally relevant and theoretically grounded. On the other hand, the results of the current research show superior psychometric properties to those documented by McCullough et al. (2002) in the creation of the Gratitude Questionnaire–Six Item Form (GQ-6). In particular, the Gratitude Toward the Organization Scale validated for the Peruvian teaching context (α between 0.89 and 0.95) surpassed the internal consistency indicators reported for the GQ-6 (α = 0.82). Furthermore, although the GQ-6 is commonly used, it was not subjected to Confirmatory Factor Analysis, nor were structural goodness of fit indices provided, which limits its usefulness as a specific tool for organizational settings. In contrast, the scale validated in this study not only preserves the theoretical basis of the concept of gratitude but also offers a more robust psychometric framework adapted for use in the educational and labor context.

Finally, the results of this study, conducted with a sample of teachers in Peru, replicate and expand the bifactorial structure proposed by Wnuk (2020) through the Gratitude Toward the Organization Scale. In terms of internal consistency, the coefficients found even showed higher values than those reported by Wnuk (2020), with the latter reporting general α = 0.90; α = 0.91 and 0.85 for each dimension. Additionally, the fit parameters of the confirmatory model in the current study (CMIN/DF = 2.872; CFI = 0.983; SRMR = 0.028; RMSEA = 0.064; *P*close = 0.031) show an excellent or acceptable fit, which is consistent with Wnuk's original findings, who documented values of CFI = 0.993; RMSEA = 0.038; NFI = 0.980; GFI = 0.976; and CMIN/DF = 1.57. These concordances and improvements indicate that the theoretical framework of the Gratitude Toward the Organization Scale is both replicable and robust, even across sociocultural contexts. However, the results suggest that the adoption of gratitude toward the organization, particularly in the educational field, may exhibit higher levels of internal consistency, likely due to the particular characteristics of the teaching work environment, such as a greater sense of community and vocation. This evidence supports the cross-cultural applicability of the scale proposed by Wnuk (2020). It suggests that it is useful for measuring organizational gratitude as both a moral (MOR) and relational (COM) attitude, with significant theoretical and practical implications for organizational climate management, which are detailed in the following sections.

### 5.1. Theoretical and practical contributions

The adaptation and validation of an organizational gratitude scale, conducted with Regular Basic Education teachers in Peru, represents a significant theoretical and practical contribution to the field of organizational studies in the country. From a theoretical perspective, this scale contributes to bridging the current gap in the scientific literature, as it is the first adaptation to Spanish and the Peruvian context. This will facilitate researchers addressing organizational gratitude in Latin American contexts, considering the particularities and nuances of Peruvian society.

Furthermore, from a practical perspective, this scale is seen as a useful and valid tool that can be used by senior management in the education sector and easily adopted by other business sectors to evaluate the effectiveness of organizational policies and practices that contribute to an optimal work environment. In this sense, its adaptation to Latin American Spanish will allow for easy application to various Latin American environments, enabling comparative analysis. Given the lack of similar studies in Peru, this pioneering research not only provides a solid theoretical framework but also paves the way for future research on the link between organizational gratitude and other aspects of performance, job satisfaction, psychological capital, perceived organizational justice, and organizational dehumanization, as well as emotional wellbeing in the Peruvian educational context.

Finally, a practical contribution also focuses on the academic community, as researchers in areas such as administration, business, and personnel management will have access to a validated, short, and easy-to-use tool for conducting future empirical studies. Business academies, research groups and networks, institutes, and universities will have the opportunity to apply the organizational gratitude scale in various practices, research projects, or institutional evaluations. It will also serve as a platform for future empirical, predictive, modeling, longitudinal, or mixed-approach studies.

### 5.2. Limitations and future research

Despite the valuable contributions offered by the adaptation and validation of the gratitude scale toward the organization in the Peruvian context, it is essential to recognize some limitations inherent to the methodology used. Firstly, the convenience sample of teachers belonging to the private educational network could limit the generalization of the results to other educational and organizational contexts in Peru. Therefore, the representativeness of the Peruvian teaching population in general could be biased, which suggests caution when extrapolating the results to other educational networks and organizations. Another limitation is the possibility of response bias, as teachers who chose to participate could differ in unobserved characteristics from those who declined.

On the other hand, the exclusion of institutions other than educational entities could affect the diversity of the sample and limit the applicability of the findings to a broader range in the country. This is even though the study intentionally focused on a homogeneous sample of Peruvian teachers of regular basic education; the methodological decision responded to the need to ensure its psychometric properties, controlling the contextual variability of different sectors, and thus guaranteeing the precision in the estimation of the psychometric properties of the metric. However, the study recognizes the possibility of applying the scale in samples that include various educational networks and public educational institutions, as well as other business sectors, which would allow additional analysis of the instrument in different organizational contexts, thus enriching the future projection of this line of research.

In addition, future studies could include additional variables, such as work tenure, the type of contract, antiquity in the organization or family burden, and being considered to explore possible correlations or predictions, not only to deepen the understanding of the factors that determine gratitude towards the organization, but also to contribute to the development of contextualized institutional policies that promote more fair and emotionally committed school environments. Finally, the implementation of more rigorous sampling methods, such as random sampling, would also contribute to improving the external validity of the results.

## 6 Conclusion

Today, gratitude toward the organization has become a fundamental aspect in the lives of all employees and is of most significant interest to organizational leaders in any sector. Without a doubt, validating a scale to measure gratitude toward the organization in the Peruvian context is a crucial step toward implementing, measuring, and developing gratitude in various business sector contexts. High-performing organizations that value and foster a positive work environment tend to create beneficial and meaningful experiences in the lives of their employees and, consequently, throughout the organization, thus generating a far-reaching positive impact.

There are determining components of gratitude that are important for every employer and worker to recognize. In this sense, every employee can deliberately choose to be aware of the positive elements and factors that are associated with their work environment to generate rewarding sensations and respond with a positive, kind, and grateful spirit during the work experience. Work environments would also be beneficial if taking into account the assumption of intentional attitudes of gratitude since these could significantly impact physical and emotional wellbeing, work motivation, productivity, organizational behaviour, prosocial behaviour, performance, and others. Desirable job benefits. This contribution suggests that the levels of perception of gratitude toward the organization should be evaluated from time to time, analysed, and serve as a guide for the design of intelligent strategies, policies, and effective programs applicable to institutions in any business sector. Therefore, the purpose of this study was to translate a scale of gratitude toward the organization into Spanish, adapt and analyse its validity and reliability.

A diligent analysis of the psychometric properties of the Gratitude Toward the Organization scale, based on the theory of positive psychology, confirms that the items are organized into two factors (COM and MOR). The findings show that the Kaiser-Meyer-Olkin test reached a high level (0.944 > 0.70) and the Bartlett test reached a highly significant level (Sig = 0.000). The Covariance Structural Equation Model (CB-SEM) was used with the AMOS 24 statistical software. Confirmatory Factor Analysis provided model fit indices (2 factors) between excellent and acceptable (CMIN/DF = 2.872; CFI = 0.983; SRMR = 0.028; RMSEA = 0.064; *P* close = 0.031). It also demonstrated good internal consistency (α = 0.890 and 0.953; CR = 0.895 and 0.954; AVE = 0.682 and 0.721). The results of this study support this measurement scale as a two-dimensional tool that can be used in business practice to measure gratitude in organizations and study the effectiveness of interventions related to wellbeing and gratitude in the workplace.

By providing academia with a Gratitude Toward the Organization measurement scale with reliable psychometric properties, adapted in practical language and promising to be agile in its application, this study could be considered an advantageous tool that contributes significantly to the science of business, to researchers of organizational behaviour, and Human Talent Management.

## Data Availability

The raw data supporting the conclusions of this article will be made available by the authors, without undue reservation.
